# Comparing appropriateness of antibiotics for nursing home residents by setting of prescription initiation: a cross-sectional analysis

**DOI:** 10.1186/s13756-018-0364-7

**Published:** 2018-06-14

**Authors:** Michael Pulia, Michael Kern, Rebecca J. Schwei, Manish N. Shah, Emmanuel Sampene, Christopher J. Crnich

**Affiliations:** 1 BerbeeWalsh Department of Emergency Medicine, University of Wisconsin-Madison School of Medicine and Public Health, Madison, WI USA; 2 University of Wisconsin-Madison School of Medicine and Public Health, Madison, WI USA; 3 Department of Biostatistics and Medical Informatics, University of Wisconsin-Madison School of Medicine and Public Health, Madison, WI USA; 4 Department of Medicine, University of Wisconsin-Madison School of Medicine and Public Health, Madison, WI USA

**Keywords:** Antibiotic stewardship, Antimicrobial resistance, Emergency department, Long-term care, Nursing home, Outpatient clinic

## Abstract

**Background:**

The pervasive, often inappropriate, use of antibiotics in healthcare settings has been identified as a major public health threat due to the resultant widespread emergence of antibiotic resistant bacteria. In nursing homes (NH), as many as two-thirds of residents receive antibiotics each year and up to 75% of these are estimated to be inappropriate. The objective of this study was to characterize antibiotic therapy for NH residents and compare appropriateness based on setting of prescription initiation.

**Methods:**

This was a retrospective, cross-sectional multi-center study that occurred in five NHs in southern Wisconsin between January 2013 and September 2014. All NH residents with an antibiotic prescribing events for suspected lower respiratory tract infections (LRTI), skin and soft tissue infections (SSTI), and urinary tract infections (UTI), initiated in-facility, from an emergency department (ED), or an outpatient clinic were included in this sample. We assessed appropriateness of antibiotic prescribing using the Loeb criteria based on documentation available in the NH medical record or transfer documents. We compared appropriateness by setting and infection type using the Chi-square test and estimated associations of demographic and clinical variables with inappropriate antibiotic prescribing using logistic regression.

**Results:**

Among 735 antibiotic starts, 640 (87.1%) were initiated in the NH as opposed to 61 (8.3%) in the outpatient clinic and 34 (4.6%) in the ED. Inappropriate antibiotic prescribing for urinary tract infections differed significantly by setting: NHs (55.9%), ED (73.3%), and outpatient clinic (80.8%), *P* = .023. Regardless of infection type, patients who had an antibiotic initiated in an outpatient clinic had 2.98 (95% CI: 1.64–5.44, *P* < .001) times increased odds of inappropriate use.

**Conclusions:**

Antibiotics initiated out-of-facility for NH residents constitute a small but not trivial percent of all prescriptions and inappropriate use was high in these settings. Further research is needed to characterize antibiotic prescribing patterns for patients managed in these settings as this likely represents an important, yet under recognized, area of consideration in attempts to improve antibiotic stewardship in NHs.

## Background

The pervasive, often inappropriate, use of antibiotics in healthcare settings has been identified as a major public health threat due to the resultant widespread emergence of antibiotic resistant bacteria [1]. In nursing homes (NH), as many as two-thirds of residents receive antibiotics each year and up to 75% of these are estimated to be inappropriate [2, 3]. This inappropriate use is in spite of existing guidelines released by the Infectious Disease Society of America that provide specific recommendations on how to evaluate and treat infection in NHs [4]. As a result, NHs can serve as reservoirs for resistant bacteria within a community [3, 5, 6].

Although commensurate attention has been given to improving antibiotic stewardship within NHs [7–10], little is known about the antibiotic prescribing for NH residents initiated in outpatient clinics or the emergency department (ED). For example, although at least 25% of NH residents visit the ED each year, the contribution and appropriateness of outpatient antibiotic therapy initiated in this setting is unknown [11]. Recent regulatory changes by the Centers for Medicare and Medicaid Services, a center within the United States Department of Health and Human Services that regulates NHs, now mandate antibiotic stewardship programs in NHs [12] and this raises an interesting dilemma for how to approach antibiotics initiated by outside providers. Understanding the burden of outside antibiotic prescribing and what documentation is required by the NH to justify ongoing treatment will be critical to guide future antibiotic stewardship efforts in this setting. As such, the aims of this study were to characterize the initiation of antibiotic therapy for NH residents by setting, infection type, and antibiotic class and then compare prescribing patterns and appropriateness between settings from the NH perspective.

## Methods

### Study design and setting

We conducted a detailed medical record extraction involving all NH residents at five southern Wisconsin facilities who had an antibiotic prescribing event from January 2013 through September 2014. Antibiotic events were identified from NH facility pharmacy records. Outpatient clinic and ED health records were reviewed when available. The location of the antibiotic start was determined by review of orders (which includes prescriptions sent from outpatient clinics or the ED), transfer documents, and documentation in the NH records. These NH facilities are required to document an assessment of any resident change-in-condition, regardless of whether they are transferred to another care setting. The change-in-condition documentation in ideal circumstances includes a detailed record of the signs and symptoms of the resident. The decision to seek care for a NH resident at an outpatient clinic or the ED could result from a wide variety of scenarios that range from patient or family member request to an acute change in condition that the NH cannot manage to routine follow up after a procedure. Most commonly this decision results from a shared decision making process that occurs between the NH staff, the resident, the resident’s family and the resident’s primary care provider.

All facilities were skilled nursing facilities located in Wisconsin, a state in the Midwest United States. Four of the facilities were located in Dane County which is the second largest County in Wisconsin with a total population of just over half a million residents and has 19 NHs [13]. One of the facilities was located in Rock County, the ninth largest County in Wisconsin with a population of 162,000 and 10 NHs [13]. The NHs in this sample had a mixture of long-term stay and post-acute care beds, and had an average of 106 beds ranging from 71 to 184. Four of the facilities had on-site nurse practitioners during regular hours and none of the facilities had a formal antibiotic stewardship program during the study period. While several of the facilities were part of a long-term care system that provided assisted living, we did not collect data in any assisted living facilities. At the time of data collection, none of these facilities used electronic prescribing and standard practice was to enact prescription orders from outpatient settings without requiring approval from a facility-associated provider. We selected facilities based on existing collaborations with the study investigators.

A trained research specialist entered health record abstraction data directly into an electronic, standardized report form in a REDCap™ database. In order to ensure consistency, the principle investigator abstracted the first 20 records and no discrepancies were observed. The local Health Sciences Minimal Risk Institutional Review Board approved all study activities.

### Participants

**Fig. 1:**
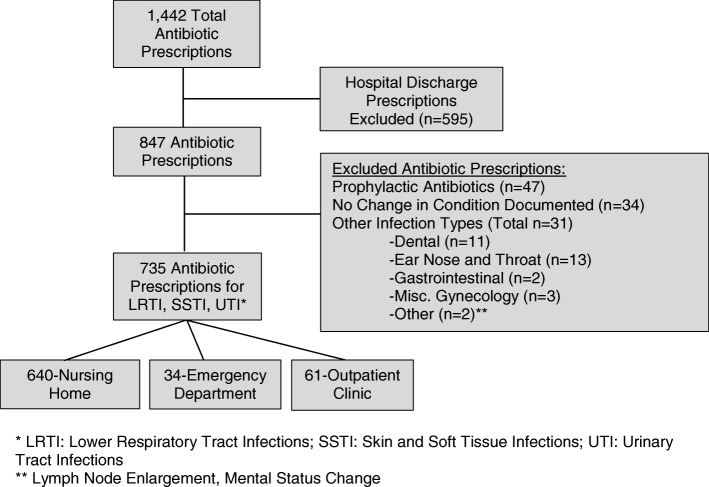
Antibiotic prescribing event flow chart

Figure 1 is a flow chart describing how the final sample was determined**.** There were 1442 antibiotics initiated in the sample. As our focus was on comparing antibiotic starts in a NH facility with antibiotic starts in an outpatient clinic or the ED, we excluded prescriptions initiated at the time of discharge from an inpatient hospital unit as noted in the NH pharmacy order records. In order to focus on the most commonly encountered bacterial infections, we only included antibiotic starts associated with a diagnosis of LRTI, SSTI, or UTI. We also excluded prophylactic antibiotic prescriptions as they are not associated with change of condition documentation and the selected appropriateness criteria is specific for acute infections.

### Methods

Data extracted included setting of prescription initiation (NH, ED, outpatient clinic), indication for antibiotic (LRIT, SSTI, UTI), patient demographics (gender, age), vital signs on day of prescription, symptoms, and antibiotic prescribed. Antibiotic appropriateness (yes, no) was determined according the Loeb consensus criteria which proposes a minimum set of clinical criteria (symptoms and vital signs) that should be present before initiating empiric antibiotics for suspected, acute bacterial infections in NH residents [14].

For the purposes of this analysis, the 15% of patients who met 2 of 4 systemic inflammatory response syndrome (SIRS) [15] criteria or 2 of 3 quick Sepsis Related Organ Failure Assessment (qSOFA) [16] criteria were considered septic regardless of prescription setting. Although there is ongoing controversy about the optimal criteria for early sepsis determination, SIRS is currently utilized in the Centers for Medicare and Medicaid Services (CMS) quality reporting measure, SEP-1, which mandates antibiotic administration within 3 h of a patient meeting severe sepsis criteria in the ED [17]. Without access to additional clinical records to enable a more definitive determination about the presence of sepsis, all antibiotic starts that met these definitions of sepsis were characterized as appropriate. Failure to take this approach would inappropriately penalize antibiotic starts in settings with a higher relative percentage of systemically ill patients who meet federally defined sepsis criteria.

### Data analysis

Differences in the frequency distributions of baseline covariates by setting were compared via Chi-square test for categorical variables and a one-way analysis of variance (ANOVA) for continuous variables. Covariates considered for all statistical approaches included age, gender, setting of antibiotic initiation and infection type. Frequency of antibiotic use by class was calculated and then ranked from most prescribed to least prescribed by setting and within each of the three infection types. The frequency of inappropriate antibiotic use by infection type and setting was also calculated.

A multivariable logistic regression was then performed to determine if any of the covariates were associated with inappropriate antibiotic use. The analytic strategy for selecting the final model was to investigate each predictor to the outcome through a univariate analysis process. After gaining inferences from the univariate analysis, interactions among the predictors were checked before proceeding to fit a full model. As the presence of sepsis criteria automatically resulted in antibiotic prescribing being characterized as appropriate, it was removed from the model due to collinearity. Since the interaction terms were not significant, our main effect model was used as our final model. All analyses are interpreted as odds ratios. Data analysis was preformed using *Stata Statistical Software: Release 14 (*College Station, TX: StataCorp LP).

## Results

Table 1 describes the patient demographics and infection type distribution of our sample across the different settings. The mean age ± SD was 84.8 ± 9.9 years and with a majority of females (71.2%). There was a significant difference in the age of patients treated by setting, with slightly younger patients managed in the ED and outpatient clinics (*p* = .006). The majority of antibiotic prescriptions were initiated within the NH (85.2%). Overall, urinary tract infection (UTI) was the most commonly treated type of infection (49.7%). Lower respiratory tract infections comprised a significantly higher proportion of cases managed in the NH (*p* = .013) while SSTI comprised a higher proportion of cases managed in the ED and outpatient clinic settings (*p* < .001). We could not calculate SIRS or qSOFA scores for 2.5% (*n* = 18) of all subjects due to missing vital sign documentation.

**Table 1:** Characteristics of nursing home antibiotic prescriptions by infection type and location

	Overall (*n* = 735)		NH (*n* = 640)		ED (*n* = 34)		Clinic (*n* = 61)		*P*-Value*
	n	%	n	%	n	%	n	%	
Mean Age, (SD)	84.8 ± 9.9		85.2 ± 9.9		83.5 ± 10.4		81.1 ± 9.8		0.006^†^
Female	523	71.2	459	71.7	25	73.5	39	63.9	0.491
Lower Respiratory Tract	195	26.5	181	28.3	7	20.6	7	11.5	0.013^†^
Skin and Soft Tissue	175	23.8	135	21.1	12	35.3	28	45.9	< 0.001^‡^
Urinary Tract	365	49.7	324	50.6	15	44.1	26	42.6	0.394
Sepsis Criteria Met	109	14.8	99	15.5	5	14.7	5	8.2	0.327

*NH*, Nursing Home; *ED*, Emergency Department

*p-value tests for independence between covariate and location of antibiotic initiation

^†^Significant at *p* < .05

^‡^Significant at *p* < .001

Inappropriate antibiotic use by infection type and setting is displayed in Table 2. Across all settings and infection types, 48.8% of antibiotic prescriptions were deemed inappropriate. This included 58.4% of UTIs, 50.7% of lower respiratory tract infections and 26.9% of skin and soft tissue infections. Overall, inappropriate antibiotic use varied significantly by setting: NH (47.5%), ED (47.1%), and outpatient clinics (63.9%), *P* = .048. Inappropriate antibiotic prescribing for UTIs varied significantly by setting: NH (55.9%), ED (73.3%), and outpatient clinics (80.8%), *P* = .023. Inappropriate antibiotic prescribing for skin and soft tissue infections also varied significantly by setting: NH (21.5%), ED (25.0%), and clinics (53.6%), *P* = .002.

**Table 2:** Inappropriate antibiotic use stratified by location of antibiotic initiation and infection type (*n* = 735)

	Overall		NH		ED		Clinic		*P*-Value*
	n	%	n	%	n	%	n	%	
Inappropriate use across all Infection Types	359	48.8	304	47.5	16	47.1	39	63.9	0.048
Inappropriate for Lower Respiratory Tract Infections	99	50.7	94	51.9	2	28.6	3	42.9	0.437
Inappropriate for Skin and Soft Tissue Infections	47	26.9	29	21.5	3	25.0	15	53.6	0.002
Inappropriate for Urinary Tract Infections	213	58.4	181	55.9	11	73.3	21	80.8	0.023

*p-value tests for independence between covariate and location of antibiotic initiation

*NH* Nursing Home, *ED* Emergency Department

A detailed representation of antibiotic class prescribing frequency by setting and infection type is available in Table 3. Fluoroquinolones were the most commonly prescribed class of antibiotic for UTIs (35.6%) and lower respiratory tract infections (34.9%). Cephalosporins were the most often prescribed class for skin/soft tissue infections (61.7%). The ED and outpatient clinics utilized a higher frequency of fluoroquinolones for lower respiratory tract infections and UTIs as compared to the NHs.

**Table 3:** Frequency of antibiotic classes prescribed by setting and infection type (*n* = 735)

	Overall		NH		ED		Clinic	
	n	%	n	%	n	%	n	%
Lower Respiratory Tract Infection	*n* = 195		*n* = 181		*n* = 7		*n* = 7	
Fluoroquinolones	68	34.9	60	33.2	5	71.4	3	42.9
Macrolides and Lincosamides	66	33.9	65	35.9	1	14.3	0	0
Penicillins and Beta-Lactamase	27	13.9	25	13.8	0	0	2	28.6
Cephalosporins	24	12.3	22	12.2	1	14.3	1	14.3
Tetracyclines	8	4.1	8	4.4	0	0	0	0
Other^a^	2	1.0	1	0.6	0	0	1	14.3
Skin/Soft Tissue	*n* = 175		*n* = 135		*n* = 12		*n* = 28	
Cephalosporins	108	61.7	87	64.4	8	66.7	13	46.4
All Penicillins/Beta-Lactamase	26	14.9	19	14.1	3	25.0	4	14.3
Fluoroquinolones	16	9.1	12	8.9	0	0	4	14.3
Tetracyclines	11	6.3	8	5.9	0	0	3	10.7
Macrolides and Lincosamides	8	4.6	4	3.0	0	0	4	14.2
Other^b^	6	3.4	5	3.7	1	8.3	0	0
Urinary Tract Infection	*n* = 365		*n* = 324		*n* = 15		*n* = 26	
Fluoroquinolones	130	35.6	112	34.6	8	53.3	10	38.5
Sulfonamides	78	21.4	73	22.5	1	6.7	4	15.4
Nitrofurantoin	66	18.1	59	18.2	2	13.3	5	19.2
Cephalosporins	58	15.9	50	15.4	3	20.0	5	19.2
All Penicillins/Beta-Lactamase	27	7.4	24	7.4	1	6.7	2	7.7
Other^c^	6	1.6	6	1.9	0	0	0	0

^a^Sulfonamides

^b^Sulfonamides, Metronidazole, Carbapenems

^c^Tetracyclines, Metronidazole, Aminoglycosides, Fosfomycin

In our multivariate analysis (Table 4), odds of inappropriate antibiotic prescribing did not vary based on each increased year of patient age or by gender. Patients who had an antibiotic initiated in an outpatient clinic had 2.98 (95% CI: 1.64–5.44) times increased odds of inappropriate use compared to antibiotic initiation in a NH, when controlling for other variables. Overall, there was a significant difference in odds of inappropriate antibiotic use by infection type. Patients with lower respiratory tract infection had 3.41 times increased odds (95% CI: 2.15–5.40) and patients with UTI had 4.47 times increased odds (95% CI: 2.96–6.77) of an inappropriate prescription when compared to SSTIs, when controlling for other variables.

**Table 4:** Odds of inappropriate antibiotic use in a logistic regression model

		Model *n* = 735 Odds Ratio (95% CI)
Age		1.00 (0.98–1.01)
Female		1.25 (0.90–1.75)
Location Antibiotic Initiated		
Nursing Home		Ref
Emergency Department		1.17 (0.56–2.44)
Outpatient Clinic		2.98 (1.64–5.44)*
Infection Type		
Skin/Soft Tissue		Ref
Lower Respiratory Tract		3.41 (2.15–5.40)*
Urinary Tract		4.47 (2.96–6.77)*

*Significant at *p* < .001

## Discussion

Through this cross sectional study of antibiotic prescribing events for NH residents, we have identified important differences in prescribing patterns between those initiated in-facility and other outpatient settings (clinic, ED) for this vulnerable population. Consistent with previously published data, overall we observed nearly 50% inappropriate antibiotic initiation for NH residents using the Loeb consensus criteria [3, 14]. Although this study was limited to 5 facilities in the state of Wisconsin, it does indicate the persistent nature of the challenge to improve judicious antibiotic use in NHs, even in the setting of increased efforts by the Centers for Disease Control (CDC) to address this issue [18]. Although the vast majority of antibiotic courses were initiated within the NHs themselves, nearly 13% (1 of 8) were initiated in either outpatient clinics or the ED. This highlights the need to consider interventions to improve prescribing not only within NHs, but also when these residents receive care outside of the facility.

When examining inappropriate prescribing in our regression model, prescriptions in the outpatient clinic setting were observed to have nearly 3 times the odds of being inappropriate. There were no increased odds with prescriptions initiated in the ED setting, which is consistent with results from several recently published reports [19–21]. Although our data do not provide insight into why the outpatient clinics might be particularly high risk for inappropriate antibiotic initiation, we hypothesize it is related to the well-established challenges associated with diagnosing acute infections in elders [22]. Providers in outpatient clinics and the ED may be significantly disadvantaged as they are often not familiar with a patient’s baseline (e.g. chronic venous stasis dermatitis mimicking cellulitis) and are not afforded time to observe the individual’s clinical trajectory that might emerge from serial examinations. However, ED providers have universal access to rapid diagnostic testing which can provide objective data and reduce diagnostic uncertainty (e.g. chest radiographs and urinalysis with microscopy). This finding highlights the need for increased efforts to establish outpatient clinic antimicrobial stewardship programs based on the CDC’s recently published guidelines for this setting [23].

Another potential factor in the observed setting variability is disagreement between medical specialties when it comes to diagnosing infections in older adults. Caterino et al. observed that nearly 20% of ED patients admitted with suspected infection were not diagnosed as such by the inpatient physicians [24]. Additionally, emergency physicians were found to over diagnose pulmonary infections and underdiagnose UTIs relative to the inpatient team’s determination [24]. This important finding highlights the need for interdisciplinary collaboration to enhance consensus in terms of diagnostic criteria and appropriate initiation of empiric antibiotics.

In addition to setting, infection type was also significantly associated with increased odds of inappropriate prescribing. UTIs increased the odds of inappropriate prescribing by nearly 4.5 times, which is consistent with prior literature highlighting the clinical uncertainty which surrounds the diagnosis of UTI in elders [24–27]. The vast majority of UTI related antibiotics were initiated in-facility with lower rates of inappropriate prescribing as compared to the ED and outpatient clinics. This perhaps reflects uptake of published methods to improve antibiotic prescribing for suspected UTI among NH residents [28]. There is also emerging evidence to suggest UTIs are often misdiagnosed in the ED setting resulting in unnecessary empiric antibiotic administration [29, 30]. A recent cohort study of elders admitted through the ED to general medical services found that 62% underwent urinalysis at the time of admission while 84% of these patients did not have symptoms of UTI [31]. Overuse of diagnostic testing, such as urinalysis, in the ED has recently come to the forefront of national discussions and certainly may play a role in the observed overtreatment of UTIs in this setting [32]. However, any attempt to improve diagnostic testing in the ED must consider both provider and system level factors such as protocol driven ordering of urinalysis in triage by nurses prior to physician evaluation [33].

Similar to UTI, LRTIs increased the odds of inappropriate prescribing by nearly 3.5 times in our regression model. This again reflects the diagnostic challenges specific to LRTIs in the older adult population. Previous literature suggests that nearly 75% of elders with pneumonia will not have fever and less than half report cough, with increased risk of atypical presentations among NH residents specifically [34–40]. Although the Loeb criteria for suspected LRTI offers five distinct sets of signs and symptoms to meet the minimum criteria to initiate antibiotics, we observed high rates of prescribing outside of these parameters. This is likely to indicate ongoing clinical concerns for atypical presentations among this high-risk population. The vast majority of LRTIs were managed in the NHs which is potentially indicative of practice patterns informed by a published clinical pathway that demonstrated similar clinical outcomes for NH residents with LRTIs treated in-facility as compared to those who were hospitalized [41].

Relative to UTIs and LRTIs, SSTIs had the lowest overall rate of inappropriate antibiotic prescribing and were therefore selected as the reference condition in the regression model. This is likely because the Loeb criteria for initiating of antibiotics for SSTIs focus more on signs of infection which are readily identified by conducting a basic physical exam as opposed to patient reported symptoms [14]. Despite this finding, antibiotic stewardship in the management of SSTIs should remain an area of great concern. This is emphasized by a recent study reporting that nearly 50% of empiric antibiotic use among NH residents with suspected SSTI failed to meet the Loeb criteria [42]. SSTIs made up a higher relative percentage of infections managed in the ED and outpatient clinics. Although the reason for this is unclear, we hypothesize that NH residents may be sent to these settings for advanced imaging if clinical uncertainty exists around the presence of a purulent SSTI. In addition, purulent SSTIs often require surgical drainage for source control, which may necessitate transfer out of the NH.

In comparing the classes of antibiotics between settings (Table 3), we observed areas of consistency and variation in practice patterns. The most common class of antibiotic used for UTIs in each care setting was fluoroquinolones, with the ED having the highest prescribing rate (53.3%). However, the second most common class used for UTIs differed by setting with NHs favoring sulfonamides while the ED and outpatient clinics favoring cephalosporins. Interestingly, these results conflict with a prior report out of Canada indicating nitrofurantoin was the most commonly used agent for treatment of UTIs in the NH setting [2]. Fluoroquinolones were also the most common class of antibiotic used for LRTIs, with the ED having the highest prescribing rate. Cephalosporins and penicillins were the two most common classes of antibiotics used for SSTIs in all settings. The observed variability in antibiotic prescribing by class again points to the need for enhanced bidirectional communication between NHs and other care settings around local resistance patterns and best practice guidelines in terms of recommended first line empiric antibiotics.

This study has several important limitations that we would like to mention. First, this study was conducted using data from 5 NHs in one US state which limits generalizability. Local practice patterns vary which highlights the need for additional investigation into antibiotic prescribing appropriateness for NH residents by setting. The small number of antibiotic starts occurring in the ED and outpatient clinic setting as compared to the NHs is also a limitation. The small sample size may have reduced our ability to detect true differences in appropriateness when present. Another limitation of this study is that our reported inappropriateness rates are likely underestimates because we did not assess the appropriateness of antibiotic selection or duration. In contrast, although we reviewed all clinical records available at the NH, documentation of care provided at outside settings (ED or outpatient clinic) was not always available. Without reviewing these records directly, it is unknown if additional symptom documentation or diagnostic testing would have satisfied the criteria for antibiotic initiation. However, our approach accurately reflects appropriateness assessed from the NH perspective based on all records available to providers in that setting. The bidirectional transfer of information for NH residents treated in the ED has already been highlighted as an important area of focus to improve the quality of care in this population [43, 44]. Although this quality concern is not specific to antibiotics, the transfer of information that enables the receiving NHs to understand the rationale for initiation of antibiotics is critical to support ongoing stewardship programs and enhancing safe transitions of care.

## Conclusions

In conclusion, our report represents the first comparative analysis of antibiotic use for NH residents based on setting of prescription initiation. Antibiotics initiated in the ED and outpatient clinics constitute a small but not trivial percent of all NH prescriptions and inappropriate use was high across all settings. Inappropriate antibiotic prescribing overall and by condition varied significantly by setting. Overall, outpatient clinics had significantly higher odds of inappropriate antibiotic prescribing, as compared to NHs and the ED. Antibiotic prescribing for NH residents that is initiated outside of the facility is an important area for additional investigation and must be considered in quality improvement efforts targeting antibiotic stewardship in NHs.

## References

[1] Ranji SR, Steinman MA, Shojania KG, Sundaram V, Lewis R, Arnold S, et al. Closing the quality gap: a critical analysis of quality improvement strategies (Vol. 4: antibiotic prescribing behavior). Rockville (MD): Agency for Healthcare Research and Quality (US); 2006. 20734528

[2] Daneman N, Gruneir A, Newman A, Fischer HD, Bronskill SE, Rochon PA (2011). Antibiotic use in long-term care facilities. J Antimicrob Chemother.

[3] Rhee SM, Stone ND (2014). Antimicrobial stewardship in long-term care facilities. Infect Dis Clin N Am.

[4] High KP, Bradley SF, Gravenstein S, Mehr DR, Quagliarello VJ, Richards C (2009). Clinical practice guideline for the evaluation of fever and infection in older adult residents of long-term care facilities: 2008 update by the Infectious Diseases Society of America. Clin Infect Dis Off Publ Infect Dis Soc Am.

[5] Augustine S, Bonomo RA (2011). Taking stock of infections and antibiotic resistance in the elderly and long-term care facilities: a survey of existing and upcoming challenges. Eur J Microbiol Immunol.

[6] Crnich CJ, Duster M, Hess T, Zimmerman DR, Drinka P (2012). Antibiotic resistance in non–major metropolitan skilled nursing facilities: prevalence and Interfacility variation. Infect Control Hosp Epidemiol.

[7] Crnich CJ, Jump R, Trautner B, Sloane PD, Mody L (2015). Optimizing antibiotic stewardship in nursing homes: a narrative review and recommendations for improvement. Drugs Aging.

[8] Dyar OJ, Pagani L, Pulcini C (2015). Strategies and challenges of antimicrobial stewardship in long-term care facilities. Clin Microbiol Infect.

[9] Nicolle LE (2014). Antimicrobial stewardship in long term care facilities: what is effective?. Antimicrob Resist Infect Control.

[10] The Core Elements of Antibiotic Stewardship for Nursing Homes | Nursing Homes and Assisted Living (LTC) | CDC n.d. https://www.cdc.gov/longtermcare/prevention/antibiotic-stewardship.html. Accessed 16, Feb 2018.

[11] Dwyer R, Gabbe B, Stoelwinder JU, Lowthian J (2014). A systematic review of outcomes following emergency transfer to hospital for residents of aged care facilities. Age Ageing.

[12] Centers for Medicare & Medicaid Services. 2016–09-28. CMS Final Improv Care Saf Consum Prot Long-Term Care Facil Resid 2016. https://www.cms.gov/Newsroom/MediaReleaseDatabase/Press-releases/2016-Press-releases-items/2016-09-28.html?DLPage=1&DLEntries=10&DLSort=0&DLSortDir=descending. Accessed 20, Apr 2018.

[13] Department of Health Services. Directory of Licensed Wisconsin Nursing Homes-Alphabetical By County and City 2018. https://www.dhs.wisconsin.gov/guide/nhdir.pdf. Accessed 20, Apr 2018.

[14] Loeb M, Bentley DW, Bradley S, Crossley K, Garibaldi R, Gantz N (2001). Development of minimum criteria for the initiation of antibiotics in residents of long-term–care facilities: results of a consensus conference. Infect Control Hosp Epidemiol.

[15] Levy MM, Fink MP, Marshall JC, Abraham E, Angus D, Cook D (2003). 2001 SCCM/ESICM/ACCP/ATS/SIS international Sepsis definitions conference. Crit Care Med.

[16] Freund Y, Lemachatti N, Krastinova E, Laer MV, Claessens Y-E, Avondo A (2017). Prognostic accuracy of Sepsis-3 criteria for in-hospital mortality among patients with suspected infection presenting to the emergency department. JAMA.

[17] Pulia MS, Redwood R, Sharp B (2017). Antimicrobial stewardship in the Management of Sepsis. Emerg Med Clin North Am.

[18] Long-term Care Facilities | NHSN | CDC n.d. http://www.cdc.gov/nhsn/ltc/. Accessed 16, Feb 2018.

[19] Barlam TF, Soria-Saucedo R, Cabral HJ, Kazis LE (2016). Unnecessary antibiotics for acute respiratory tract infections: association with care setting and patient demographics. Open Forum Infect Dis.

[20] Bergmark RW, Sedaghat AR (2016). Antibiotic prescription for acute rhinosinusitis: emergency departments versus primary care providers. Laryngoscope.

[21] Jones BE, Sauer B, Jones MM, Campo J, Damal K, He T (2015). Variation in outpatient antibiotic prescribing for acute respiratory infections in the veteran population: a cross-sectional study. Ann Intern Med.

[22] Caterino JM (2008). Evaluation and management of geriatric infections in the emergency department. Emerg Med Clin North Am.

[23] Core Elements of Outpatient Antibiotic Stewardship | Community | Antibiotic Use | CDC 2017. https://www.cdc.gov/antibiotic-use/community/improving-prescribing/core-elements/core-outpatient-stewardship.html. Accessed 16 Feb 2018.

[24] Caterino JM, Stevenson KB (2012). Disagreement between emergency physician and inpatient physician diagnosis of infection in older adults admitted from the emergency department. Acad Emerg Med.

[25] Drinka PJ, Crnich CJ (2008). Diagnostic accuracy of criteria for urinary tract infection in a cohort of nursing home residents. J Am Geriatr Soc.

[26] Juthani-Mehta M, Tinetti M, Perrelli E, Towle V, Van Ness PH, Quagliarello V (2007). Diagnostic accuracy of criteria for urinary tract infection in a cohort of nursing home residents. J Am Geriatr Soc.

[27] Nace DA, Drinka PJ, Crnich CJ (2014). Clinical uncertainties in the approach to long term care residents with possible urinary tract infection. J Am Med Dir Assoc.

[28] Loeb M, Brazil K, Lohfeld L, McGeer A, Simor A, Stevenson K (2005). Effect of a multifaceted intervention on number of antimicrobial prescriptions for suspected urinary tract infections in residents of nursing homes: cluster randomised controlled trial. BMJ.

[29] Tomas ME, Getman D, Donskey CJ, Hecker MT (2015). Over-diagnosis of urinary tract infection and under-diagnosis of sexually transmitted infection in adult women presenting to an emergency department. J Clin Microbiol.

[30] Watson JR, Sánchez PJ, Spencer JD, Cohen DM, Hains DS (2018). Urinary tract infection and antimicrobial stewardship in the emergency department. Pediatr Emerg Care.

[31] Yin P, Kiss A, Leis JA (2015). Urinalysis orders among patients admitted to the general medicine service. JAMA Intern Med.

[32] Sullivan W, Tintinalli J, Hoffman J, Kanzari H, Probst M. Pro/con: ‘unnecessary’ testing. Emergency Physicians Monthly n.d. http://epmonthly.com/article/pro-con-unnecessary-testing/. Accessed 14, May 2018.

[33] Framework for Quality and Safety in the Emergency Department n.d. https://www.ifem.cc/wp-content/uploads/2016/03/Framework-for-Quality-and-Safety-in-the-Emergency-Department-2012.doc.pdf. Accessed 16 Feb 2018.

[34] Fine JM, Fine MJ, Galusha D, Petrillo M, Meehan TP (2002). Patient and hospital characteristics associated with recommended processes of care for elderly patients hospitalized with pneumonia: results from the medicare quality indicator system pneumonia module. Arch Intern Med.

[35] Harper C, Newton P (1989). Clinical aspects of pneumonia in the elderly veteran. J Am Geriatr Soc.

[36] Marrie TJ, Haldane EV, Faulkner RS, Durant H, Kwan C (1985). Community-acquired pneumonia requiring hospitalization. J Am Geriatr Soc.

[37] Metlay JP, Schulz R, Li YH, Singer DE, Marrie TJ, Coley CM (1997). Influence of age on symptoms at presentation in patients with community-acquired pneumonia. Arch Intern Med.

[38] Muder RR, Brennen C, Swenson DL, Wagener M (1996). Pneumonia in a long-term care facility. A prospective study of outcome. Arch Intern Med.

[39] Starczewski AR, Allen SC, Vargas E, Lye M (1988). Clinical prognostic indices of fatality in elderly patients admitted to hospital with acute pneumonia. Age Ageing.

[40] Waterer GW, Kessler LA, Wunderink RG (2006). Delayed administration of antibiotics and atypical presentation in community-acquired pneumonia. Chest.

[41] Loeb M, Carusone SC, Goeree R, Walter SD, Brazil K, Krueger P (2006). Effect of a clinical pathway to reduce hospitalizations in nursing home residents with pneumonia: a randomized controlled trial. JAMA.

[42] Feldstein D, Sloane PD, Weber D, Ward K, Reed D, Zimmerman S (2017). Current prescribing practices for skin and soft tissue infections in nursing homes. J Am Med Dir Assoc.

[43] Terrell KM, Hustey FM, Hwang U, Gerson LW, Wenger NS, Miller DK (2009). Quality indicators for geriatric emergency care. Acad Emerg Med.

[44] Terrell KM, Miller DK (2006). Challenges in transitional care between nursing homes and emergency departments. J Am Med Dir Assoc.

